# Syphilis and the Nervous System

**Published:** 1893-09

**Authors:** 


*Syphilis and the Nervous System.* By W. R. GOWERS, M.D., Pp. 131. London: J. & A. CHURCHILL. 1892.

This is a reprint of Dr. Gowers's admirable Lettsomian Lectures, which we believe have been translated into several languages. They certainly cannot be too widely known, or too closely studied. The lectures gave rise to much discussion at the time of their delivery, especially as regards the truth of Dr. Gowers's statement that syphilis is incurable. In the way in which he uses the term incurable, we think that his view is undoubtedly the correct one. Especially has he done good service in pointing out that the more indirect consequences of syphilitic processes on the nervous centres cannot be remedied; that, for instance, the necrosis of brain tissue which follows occlusion of an artery is as irremovable if it arise from syphilitic as if it come from atheromatous disease. Hence the paramount importance of early treatment, and the necessity for every medical man to familiarise himself with the early symptoms of syphilis of the nervous system. This book will enable the reader to form a rational diagnosis as to the presence or not of syphilitic lesions, and will also instruct him how the various steps in the more difficult diagnosis of the exact nature and seat of the lesion present should be carried out. No subject can be of greater importance, on account of the immense benefit that may result from timely treatment, and the disastrous effects that too often occur in patients who have not been properly treated. The admirably clear and terse style of the author renders his pages pleasant reading, and the philosophic manner in which he treats his subject makes it a model of what such a book should be. No doubt the large majority of our readers will know these lectures, and will be glad to possess them in the clear print, convenient size, and neat binding of this reprint.

